# Dissociating effects of subclinical anxiety and depression on cognitive control

**DOI:** 10.2478/v10053-008-0100-6

**Published:** 2012-02-15

**Authors:** Jody Ng, Hoi Yan Chan, Friederike Schlaghecken

**Affiliations:** Department of Psychology, University of Warwick, Coventry, UK

**Keywords:** cognitive control, inhibition, subclinical depression, subclinical anxiety, hybrid masked prime-Simon task

## Abstract

Even at subclinical levels, anxiety and depression are associated with impaired cognitive control. It is unclear, though, to what extent these deficits reflect a common underlying dysfunction. Using a non-affective hybrid masked prime-Simon task, we obtained several measures of within- and between- trial inhibitory behavioral control in 80 young, healthy volunteers, together with measures of their anxiety and depression levels. Neither depression nor anxiety affected low-level within-trial control, or any of the between-trial control measures. However, increased levels of depression, but not of anxiety, were associated with impaired high-level within-trial control (increased Simon effect). Results indicate that depression, but not anxiety, impairs voluntary online response-control mechanisms independent of affective content.

## Introduction

Anxiety disorders and depression are typically associated with dysfunctional cognitive control, specifically in the form of an attentional bias towards negative information (e.g., Cisler & Koster, 2010; Peckham, McHugh, & Otto, 2010) and impaired inhibitory control (e.g., Channon & Green, 1999; Kaiser et al., 2003). Despite the high comorbidity of these two disorders (75% and above; e.g., Lamers et al., 2001), which points to a common etiology, anxiety and depression have different underlying neural correlates (e.g., Broyd et al., 2009; Liotti & Mayberg, 2001; van Tol et al., 2010). However, both are associated with structural and functional abnormalities in prefrontal cortical regions (for a recent review, see Mitchell, 2011), most notably a reduced volume of the dorsal anterior cingulate cortex (ACC; e.g., van Tol et al., 2010). This structure is widely believed to be critically involved in inhibitory cognitive control (see e.g., Carter & van Veen, 2007). Its supposed role is to detect conflict between competing neural representations in the perceptuo-motor system, and to issue a signal to the dorso-lateral prefrontal cortex (dlPFC) to adjust the system towards a more “cautious” mode.

While major depression and clinical anxiety disorders are crippling illnesses severely affecting a person’s life, some of their symptoms - like those of other disorders - are experienced in a milder form even by psychologically healthy individuals. Yet even at subclinical levels, anxiety and depression can adversely affect inhibitory cognitive control (e.g., Ansari & Derakshan, 2010, 2011; Ansari, Derakshan, & Richards, 2008; Avila & Parcet, 2002; Holmes & Pizzagalli, 2007). Similarly, like clinical anxiety and depression, their subclinical symptoms show some relationship to reduced activity within anterior cortical control structures: For instance, levels of subclinical anxiety have been found to be inversely related to dlPFC activity in a conflict task (Bishop, 2008), and there is some evidence of an inverse relationship between subclinical depression (and, to a lesser extent, between subclinical anxiety) and the resting-state activity of the ACC (Wacker, Dillon, & Pizzagalli, 2009). Finally, again like clinical anxiety and depression, elevated levels of subclinical anxiety and depression symptoms frequently occur together (Barlow & Campbell, 2000), suggesting a common underlying cause. This, however, poses a major theoretical obstacle to the interpretation of the above-mentioned findings: If anxiety and depression are, in fact, related but *separate* dysfunctions, then their frequent co-occurrence results in a substantial confound - any inhibitory deficit attributed to anxiety might have been driven by elevated levels of depression, and vice versa, if care is not taken to account for one while investigating the other. However, to the best of our knowledge, there has been no attempt yet to tease apart the respective impact of subclinical anxiety and depression levels on inhibitory cognitive control.

The present study was designed to address this issue with regard to inhibitory deficits in the perceptuo-motor domain.^1^ We were particularly interested in separating the effects of anxious or depressed affect from affective processing (i.e., the processing of emotionally valenced stimulus material). Therefore, we employed a non-affective perceptuo-motor control task using emotionally neutral stimuli. In addition, we measured participants’ level of anxiety and depression using self-assessment questionnaires.

## Experiment

### Behavioral inhibitory effects being measured

The present study employed an emotionally neutral hybrid masked prime-Simon task (see Maylor, Birak, & Schlaghecken, 2011; Schlaghecken, Refaat, & Maylor, 2011). In this task, participants have to give a spatially corresponding manual response to the direction of an arrow stimulus (e.g., left-hand response to a left-pointing arrow). Each target is presented at a (task-irrelevant) left or right-hand screen location, and is preceded by a (task-irrelevant) centrally presented, backward-masked arrow prime. Prime identity and target location randomly and independently match or mismatch the required response. On prime-compatible trials, prime and target are associated with the same response, on prime-incompatible trials, they are associated with opposite responses. On location-congruent trials, response hand and target location match (e.g., a left-pointing arrow, requiring a left-hand response, appears on the left-hand side of the screen); on location-incongruent trials, they mismatch (e.g., a left-pointing arrow, requiring a left-hand response, appears on the right-hand side). This paradigm allows us to measure a number of behavioral inhibitory effects:

#### Negative compatibility effect (NCE)

Although masked primes are inaccessible to conscious awareness (e.g., Eimer & Schlaghecken, 2002; Schlaghecken, Birak, & Maylor, 2011), they systematically affect responses to the target, with slower and more error-prone responses on prime-compatible than on prime-incompatible trials. The NCE has been interpreted as reflecting a fast inhibition of the response tendency initially triggered by the prime (Boy, Clarke, & Sumner, 2008; Jaśkowski, 2008; Jaśkowski & Przekoracka-Krawczyk, 2005; Schlaghecken & Eimer, 2002, 2006; Schlaghecken, Klapp, & Maylor, 2009; Schlaghecken, Rowley, Sembi, Simmons, & Whitcomb, 2007; Sumner, 2008): If the target requires the same response as the prime, this just-inhibited response has to be re-activated, resulting in longer response latencies, whereas if the target requires the opposite response, the non-inhibited (and possibly even disinhibited; cf. Schlaghecken, Bowman, & Eimer, 2006) response can be executed quickly and accurately. A notable feature of the NCE is that in young, healthy adults, it develops very quickly, reaching its peak at masked prime-target intervals of 150-200 ms (e.g., Schlaghecken, Birak, & Maylor, 2011; Sumner & Brandwood, 2008). In older adults, however, it is virtually absent within this time window (Maylor et al., 2011; Schlaghecken & Maylor, 2005).^2^ The effects of depression on perceptuo-motor control bear some similarity to those of normal aging (e.g., Seidler et al., 2010). To the extent that this holds for subclinical depressive symptoms as well, one might expect that in the present study, elevated levels of depression are associated with reduced or even absent NCEs.^3^

#### Simon effect

Responses are typically faster on location-congruent than on location-incongruent trials, as an incorrect response tendency - triggered automatically by the incongruent target location - has to be overcome before a correct response can be executed (e.g., Stürmer, Siggelkow, Dengler, & Leuthold, 2000). Consequently, the magnitude of the Simon effect represents a measure of the strength of inhibitory control: In a system that deals efficiently with the interfering response tendency, location-incongruent responses will only be slightly delayed, whereas in a system with inefficient interference suppression, they will be substantially delayed.^4^ Thus to the extent that subclinical anxiety and depression affect response inhibition, we expect larger Simon effects in participants with higher levels of anxiety and depression.

#### Gratton effect

Cognitive control is not restricted to dealing with already-present conflicts. Arguably, an even more important task is to dynamically adjust neural processing to the presence or absence of conflict in the environment, and such influences can be measured as sequential effects in response conflict paradigms like the Simon task. Simon effect magnitude varies as a function of the congruency of the preceding trial: Following a location-congruent trial, Simon effects are typically much larger than following a location-incongruent trial, where they might be absent or even reversed. This sequential modulation of interference effects is known as the *Gratton effect* (e.g., Gratton, Coles, & Donchin, 1992; Stürmer et al., 2000; Wühr & Ansorge, 2005). As a second-order effect, the relationship between Gratton effect magnitude and strength of inhibitory control is not straight-forward. On the one hand, a small Gratton effect might be due to already-small Simon effects following location-congruent trials, indicating strong inhibitory control. On the other hand, it might be due to relatively large Simon effects following location-incongruent trials, indicating weak inhibitory control. Thus the pattern of reaction times across the four trial conditions (congruent followed by congruent [cC], congruent followed by incongruent [cI], incongruent followed by congruent [iC], and incongruent followed by incongruent [iI]) will be a better indicator of the strength of inhibitory control than the Gratton effect as such. Specifically, a deficit of dynamic inhibitory adjustment should be reflected in longer iI reaction times.^5^

#### Post-error slowing (PES)

Responses are typically slower and more likely to be correct following an incorrect than following a correct response. There are various reasons for this. First, similar to the Gratton effect, PES might reflect an anticipatory adjustment: Participants might voluntarily suppress acti-vity in the perceptuo-motor system in order to minimize the chance for a subsequent error (e.g., Carter & van Veen, 2007). Second, they might not yet have overcome the processing problem that caused the error in the preceding trial (e.g., Gehring, Goss, Coles, Meyer, & Donchin, 1993). Third, the error, because of its relative rarity, might have drawn the participant’s attention away from the task at hand (e.g., Notebaert et al., 2009).^6^ For more anxious participants, committing an error might appear particularly “threatening”, whereas there is no reason to believe that the same is true for more depressed participants. Thus anxiety, but not depression, might make one particularly prone to the attention-orienting mechanism of PES, suggesting that higher levels of anxiety, but not higher levels of depression, might be associated with larger PES. This has indeed been confirmed at least for introverts (Robinson, Meier, Wilkowski, & Ode, 2007). Patients suffering from clinical depression, on the other hand, largely fail to show PES, possibly reflecting a more general state of blunted responses to environmental or feedback information (Steele, Kumar, & Ebmeier, 2007). Even at subclinical levels, participants with higher depression scores not only failed to show PES, but also failed to show the usual improved post-error accuracy (Pizzagalli, Peccoralo, Davidson, & Cohen, 2006).

#### Post-conflict slowing (PCS)

Even following a correct response, responses are typically slower following an incongruent trial than following a congruent trial (e.g., Schlaghecken, Refaat, & Maylor, 2011). This might merely reflect a “passive” self-organization mechanism (e.g., Laming, 1979; Van Orden, Holdern, & Turvey, 2005). Alternatively, however, PCS might represent a form of inhibitory context adaptation (i.e., a deliberate slowing of responses if the immediate context contained a slow response), and as such might be affected by elevated levels of anxiety or depression symptoms.

In the following, we will refer to the last four of these effects (Simon effect, Gratton effect, PES, and PCS) as reflecting “high-level” control processes: These are processes triggered by a consciously perceived conflict, and there is evidence that at least the first three are mediated by the same (or highly overlapping) anterior cortical structures (e.g., Carter & van Veen, 2007; Morishima, Okuda, & Sakai, 2010). Consequently, we will refer to the NCE as reflecting a “low-level” control process, that is, a process which (a) inhibits a motor tendency triggered by a non-consciously perceived stimulus, and which (b) appears to be mediated by basal ganglia-thalamo-cortical circuits rather than by the anterior control system (Aron et al., 2003). Furthermore, we will refer to the NCE and the Simon effect as effects reflecting “within-trial” or “online” inhibitory control, and to the Gratton effect, PES, and PCS as reflecting “between-trial” or “adaptive” inhibitory control.

### Method

#### Participants

Eighty-three students of the University of Warwick (37 male), aged 17 to 25 years (*M* = 20.3, *SD* = 1.4), participated in the experiment. All but six participants were right-handed.

#### Apparatus and Stimuli

##### Hybrid masked prime-Simon Task

Left- and right-pointing double arrows (<< and >>) served as prime and target stimuli, subtending a visual angle of approximately 1.6º × 0.7º. Masks were constructed on the basis of a virtual 9 × 9 grid, randomly filled with overlapping horizontal, vertical, and oblique lines of different lengths (none of them having the same orientation as the lines making up the arrow stimuli), resulting in a roughly rectangular array of approximately 4.6º × 2.0º. A new random mask was created on each trial in order to avoid perceptual learning of the mask and correspondingly increased prime identification (Schlaghecken, Blagrove, & Maylor, 2008; Schubö, Schlaghecken, & Meinecke, 2001). Stimuli were presented in black on white on a 15’’ computer screen.

##### Zung Self-rating Depression Scale (ZSDS) and Zung Self-rating Anxiety Scale (ZSAS)

The ZSDS (Zung, 1965) is a 20-item self-report questionnaire measuring cognitive, mood, and somatic symptoms of depression (Passik et al., 2000). Each item is rated on a 4-point Likert-type scale. The ZSDS has good reliability and validity (e.g., Dugan et al., 1998). The ZSAS (Zung, 1971) is a 20-item self-report questionnaire measuring symptoms of anxiety disorder, specifically feelings of anxiousness and panic, vestibular and gastrointestinal/muscular sensations, and somatic control (Olatunji, Deacon, Adramowitz, & Tolin, 2006). Each item is rated on a 4-point Likert-type scale. The ZSAS has good reliability and validity (e.g., Olatunji et al., 2006).

#### Procedure

**Figure 1.**
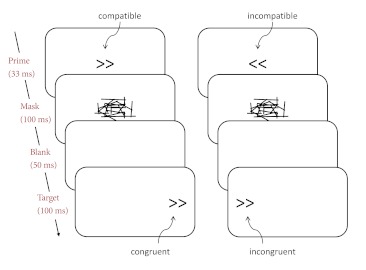
Stimulus material and trial structure. The figure depicts a compatible congruent and an incompatible incongruent trial, both requiring a right-hand response.

Participants were seated in a dimly lit room approximately 60 cm in front of a computer screen. In the first part of the experiment, they completed the hybrid masked prime-Simon task. As shown in Figure 1, each trial began with a centrally presented prime (33 ms), followed immediately by a mask (100 ms). After a 100-ms blank, a target was presented for 100 ms, approximately 14º to the left or right of fixation. Inter-trial interval was 1,460 ms. Response keys were the left and right SHIFT keys on a standard qwerty keyboard. Participants were instructed to respond as quickly and accurately as possible to the direction of each target arrow, and to ignore its location. They first completed a 24-trial practice phase, during which the experimenter remained in the room to offer further advice if necessary. Subsequently, participants completed six experimental blocks of 72 trials each. Within each block, all eight conditions (2 prime-compatibility × 2 location-congruency × 2 responses) were fully randomized and appeared with equal frequency.

Participants started each new block whenever they felt ready to do so. In the second part of the experiment, participants completed the ZSDS and the ZSAS. The experiment lasted approximately 30 min in total. Written fully informed consent was obtained prior to the experiment, and participants were reminded again at the end that they had the right to withdraw their data without explanation.

#### Data Analysis

##### Anxiety and depression scores

After reversing the scores for reverse-scored items, participants’ answers to the 20 questions in each questionnaire (from 1 for the lowest to 4 for the highest indicator of anxiety/depression) were summed. The lowest possible score for each questionnaire thus was 20, the highest was 80. For direct comparison of low- versus high-scoring participants, a median split was conducted (separately for each questionnaire), and participants who produced the exact median score were excluded from that analysis. For correlations between behavioral measures and anxiety/depression scores, all participants were entered into the analysis.

##### Reaction times (RTs)

Trials were grouped according to the preceding trial’s location-congruency (congruent, incongruent), the current trial’s location-congruency (congruent, incongruent), and the current trial’s prime-compatibility (compatible, incompatible). Taking into account only trials where both the current and the preceding response were correct, each participant’s mean RTs were calculated for these eight trial types. Additionally, post-error RTs were calculated by averaging RTs of all correct responses following an incorrect response (due to insufficient numbers of trials, this could not be done separately for individual trial types).

##### RT effects

To account of overall RT differences between participants (more than 200 ms between the fastest and the slowest responder), RT effects were calculated as ratios rather than as differences:

1. Post-conflict slowing (PCS) as the ratio of mean RT on all trials following a location-incongruent trial (“previous incongruent”, PI) to the mean RT on all trials following a location-congruent trial (“previous congruent”, PC);

2. Simon effects as the ratio of mean RT on location-incongruent trials to mean RT on location-congruent trials, separately for PC and PI trials;

3. Gratton effect as the ratio of PC Simon effect to PI Simon effect;^7^

4. NCEs as the ratio of mean RT on prime-incompatible trials to the mean RT on prime-compatible trials;

5. Post-error slowing (PES) as the ratio of post-error RTs to mean (post-correct) RTs.

##### Error rates and exclusion criteria

Because error rates were very low (< 5% on average), most statistical analyses of error rates were invalidated by floor effects. In order to at least partly overcome this problem, we collapsed across the factor prime-compatibility (for which we had no predictions regarding any interaction with either anxiety or depression scores). Thus error rates were calculated for cC, cI, iC, and iI trials, separately for trials following a correct and trials following an incorrect response.

Three participants were excluded from all statistical analyses because of insufficient numbers of valid trials (less than 10 for one or more trial type). One further participant was excluded because of excessively slow responses (overall mean RT more than 2.5 *SD*s above the group mean), leaving a sample of 80 participants.

##### Statistical analyses

RTs of the complete data set were analyzed using a repeated measures ANOVA with the within-subject factors location congruency on the Previous Trial (congruent, incongruent), Location Congruency of the current trial (congruent, incongruent), and Prime Compatibility of the current trial (compatible, incompatible). Post-error slowing was analyzed using a univariate ANOVA. Next, these analyses were repeated (a) with Anxiety (high, low) as a between-subject factor (excluding participants with median scores on the anxiety questionnaire), and (b) with Depression (high, low) as a between-subject factor (excluding participants with median scores on the depression questionnaire). Follow-up analyses were carried out in form of partial correlations between affect scores and mean RTs, and between affect scores and RT effects. When correlating with anxiety scores, depression scores were controlled for, and when correlating with depression scores, anxiety scores were controlled for.

Error rates were analyzed using a repeated-measures ANOVA with the between-subject factors Anxiety Group (high, low) and Depression Group (high, low), and the within-subject factors Previous Response (correct, incorrect), Previous Location-Congruency (congruent, incongruent), and Current Location-Congruency (congruent, incongruent).

### Results

#### Anxiety and Depression Scores

Scores on the anxiety scale (*M* = 32.8, *SD* = 6.83, range: 22-51, *Mdn* = 31) were significantly lower than scores on the depression scale (*M* = 37.6, *SD* = 7.70, range: 21-62, *Mdn* = 37), *t*(79) = 7.48, *p* < .001. Of the 80 participants, 35 scored below and 38 scored above the median anxiety score, and 39 scored below and 35 scored above the median depression score. However, even the higher-scoring groups remained well below the mid-point of the scale for both measures. As expected, anxiety and depression scores were highly correlated, *r* = .698, *p* < .001.

#### Error Rates

**Figure 2.**
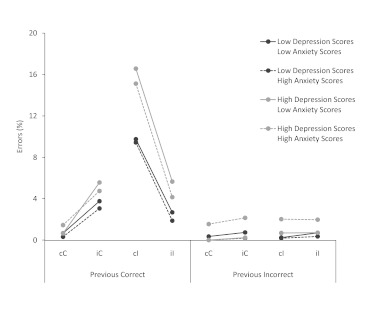
Error rates (%) as a function the four location-congruency trial types (cC = previous congruent, current congruent; cI = previous congruent, current incongruent; iC = previous incongruent, current congruent; iI = previous incongruent, current incongruent), and for errors committed after a correct response and after an incorrect response, plotted separately for participants with either low (dark grey) or high (light grey) depression scores, and low (solid lines) or high (dashed lines) anxiety scores. Error bars indicate ±1 standard error of mean.

Error rates are depicted in Figure 2. Error rates following an incorrect response were significantly lower than those following a correct response (post-error adjustment), lower following a location-incongruent trial than following a congruent trial (PCS), and lower on location-congruent than on incongruent trials (Simon effect), all *Fs*(1, 64) > 27.0, all *ps* < .001. All two- and three-way interactions between these factors (i.e., Gratton effect and post-error modulations) were also highly significant, all *Fs*(1, 64) > 45.0, all *ps* < .001.^8^ Furthermore, Depression Group interacted significantly with Current Location-Congruency, *F*(1, 64) = 6.0, *p* =.017, *MSE* = 721.90, as participants with elevated depression levels produced larger Simon effects (i.e., produced more errors on location-incongruent trials) than participants with low depression levels. There was no main effect of either Anxiety or Depression, and no other interactions with these factors, all *Fs* < 3.5, all *ps* .06.

In order to explore the effect of anxiety and depression on error rates and post-error adjustments without distortion by floor effects, we analyzed cI trials in isolation. The analysis confirmed that participants in the high-depression group produced more errors and larger post-error adjustments than participants in the low-depression group, both *Fs* > 4.2, both *ps* .043 (both effects remained when covarying anxiety scores, both *Fs* > 4.1, both *ps* .047). In contrast, anxiety levels did not affect error rates or post-error adjustments on cI trials, all *Fs* < 1 (with or without covarying depression scores).

#### Reaction times

**Figure 3.**
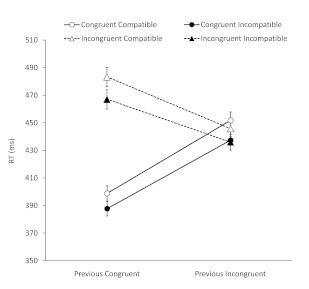
Mean reaction times (RTs) on congruent (circles, solid lines) and incongruent (triangle, dashed lines) trials, plotted separately for compatible (white) and incompatible (black) trials, and separately for trials following congruent and trials following incongruent trials. Error bars indicate ±1 standard error of mean.

Figure 3 shows mean RTs (across all participants) for each of the eight trial types. Overall, responses were faster following a location-congruent than following a location-incongruent trial (PCS), *F*(1, 79) = 63.91, *p* < .001, *MSE* = 210.09; faster with location-congruent than with location-incongruent targets (Simon effect), *F*(1, 79) = 275.40, *p* < .001, *MSE* = 899.62; and faster with prime-incompatible than with prime-compatible targets (NCE), *F*(1, 79) = 72.47, *p* < .001, *MSE* = 337.12. Simon effects following location-incongruent trials were much reduced (in fact, numerically reversed) relative to Simon effects following location congruent trials (Gratton effect), *F*(1, 79) = 376.04, *p* < .001, *MSE* = 764.47.

The two-way interactions between Previous Trial and Prime Compatibility, and between Location Congruency and Prime Compatibility, were non-significant, both *Fs* < 1; however, there was a significant three-way interaction between Previous Trial, Location Congruency, and Prime Compatibility, *F*(1, 79) = 5.98, *p* =.017, *MSE* = 113.36.

#### Effects of Anxiety and Depression on RTs: Group Analysis

**Figure 4.**
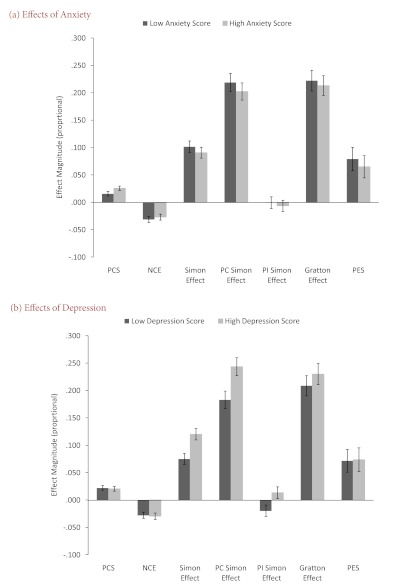
Magnitude of reaction time (RT) effects, expressed as RT ratios, plotted separately for low (dark grey) and high (light grey) scores on (a) the depression scale, and (b) the anxiety scale. PCS = post-conflict slowing (previous incongruent RT : previous congruent RT). NCE = negative compatibility effect (prime-incompatible RT : prime-compatible RT). PC Simon effect = previous congruent Simon effect (location-incongruent RT : location-congruent RT). PI Simon Effect = previous incongruent Simon Effect. Gratton effect (PC Simon effect : PI Simon effect). PES = post-error slowing. Note that for display purposes, values have been rescaled such that the baseline (no RT difference) is set a 0 rather than 1. Error bars indicate ±1 standard error of mean.

Repeating the analysis with Anxiety (low group, high group) as a between-subject factor (Figure 4, top panel) showed that the two-way interaction of Previous Congruency × Anxiety and the four-way interaction of Previous Congruency × Current Congruency × Compatibility × Anxiety approached significance, both *Fs*(1, 71) > 2.95, both *ps* < .10. However, when depression scores were entered as a covariate, these effects disappeared, both *Fs* < 2.1, both *ps* .15. No other effects of an-xiety even approached statistical significance, neither with nor without covarying depression scores, all *Fs* < 2.1, all *ps* .15.

A different picture emerged when using Depression (low group, high group) as the between-subject factor (Figure 4, bottom panel). Participants with higher depression scores showed significantly larger Simon effects than participants with low scores, *F*(1, 72) = 5.97, *p* =.017, *MSE* = 896.87, and this difference became even more pronounced when anxiety scores were entered as a covariate, *F*(1, 71) = 6.46, *p* =.013, *MSE* = 896.03. No other effects of depression were statistically significant, neither with nor without covarying anxiety scores, all *Fs* < 3.8, all *ps* > .05.

Overall post-error slowing (PES) was significant, as indicated by a one-sample *t*-test, *t*(79) = 5.44, *p* < .001, but this effect was not modulated by either anxiety or depression, all *Fs* < 1. As the error rate analysis (see above) suggested that reliable effects might be restricted to cI trials, we repeated the RT analysis for these trials separately, but obtained the same result (i.e., significant PES, but no modulation by anxiety or depression).

#### Effects of Anxiety and Depression on RTs: Correlation Analysis

To explore these effects in more detail, correlations between anxiety (depression) scores and RT measures were calculated, partialling out scores from the respective alternative scale. A first series of analyses showed that patterns of correlation were driven mainly by a trial’s (current and previous) target congruency, whereas prime compatibility did not appear to contribute. Therefore, RTs were averaged across compatible and incompatible trials.

As shown in Table 1, anxiety scores showed a weak positive correlation with RTs, which was significant for all but location-incongruent responses following an incongruent trial. In contrast, depression scores showed a weak negative correlation with RTs, which was significant only for location-congruent responses following a congruent trial. Results of the partial correlations between anxiety (depression) scores and the various measures of inhibitory control are given in Table 2.

**Table 1.** Results of Partial Correlations Between Anxiety Scores (controlled for depression scores) and Reaction Times, and of Partial Correlations Between Depression Scores (controlled for anxiety scores) and Reaction Times.

			Previous congruent		Previous incongruent	
Conrolled for			Congruent	Incongruent	Congruent	Incongruent
Depression score	**Anxiety score**	*r*	**.268**	.157	**.225**	**.259**
		*p*	**.017**	.166	**.046**	**.021**
Anxiety score	**Depression score**	*r*	**-.225**	-.070	-.158	-.135
		*p*	**.046**	.538	.165	.234
		*df*	77	77	77	77

*Note*. Bold numbers indicate significant correlations.

**Table 2.** Results of Partial Correlations Between Anxiety Scores (controlled for depression scores) and Reaction Time Effects, and of Partial Correlations Between Depression Scores (controlled for anxiety scores) and Reaction Time Effects.

Controlled for			**PCS**	**NCE**	**Overall Simon effect**	**PC Simon effect**	**PI Simon effect**	**Gratton effect**	**PES**
Depression score	**Anxiety score**	*r*	.149	.006	-.124	-.165	-.013	-.142	.046
		*p*	.191	.959	.278	.146	.908	.212	.690
Anxiety score	**Depression score**	*r*	-.030	-.002	**.221**	**.243**	.094	.157	.054
		*p*	.796	.989	**.050**	**.031**	.411	.168	.639
		*df*	77	77	77	77	77	77	77

*Note*. All reaction time effects are expressed as ratios (see Method section). PCS = post-conflict slowing. PC = previous trial congruent. PI = previous trial incongruent. PES = post-error slowing. Bold numbers indicate significant correlations.

They confirm the pattern observed in the ANOVAs: Whereas there was no systematic link between anxiety and the magnitude of any of the RT effects, depression scores correlated positively with Simon effects (particularly with Simon effects following a congruent trial).

## Discussion

The present study investigated whether subclinical levels of anxiety and depression impair inhibitory control of responses to affectively neutral visual stimuli. Specifically, we analyzed effects of anxiety while controlling for depression scores, and effects of depression while controlling for anxiety. Using a hybrid masked prime-Simon task, we measured the NCE as an index of low-level online (within-trial) inhibition; the Simon effect as an index of high-level online inhibition; and post-conflict slowing (PCS), Gratton effect, and post-error adaptation (PES and post-error error reduction) as indices of high-level adaptive (between-trial) inhibition. All of these effects were significant, showing that overall, participants were influenced by and adjusted their behavior to the different stimulus conditions.

However, behavioral modulations by elevated levels of anxiety and depression were limited, and were restricted to a small subset of measures. To some extent, this might have been due to the limited range of anxiety and depression scores: More wide-ranging effects might have been obtained with higher variability of anxiety and/or depressive symptoms. However, even with the generally low symptom levels, systematic effects could be observed. In particular, increased levels of anxiety were found to be associated with a slight increase in overall reaction times (RTs), whereas increased levels of depression were associated with enlarged Simon effects both for error rates (driven by an increased error rate on incongruent trials) and for RTs (driven mainly by decreased RTs on congruent trials preceded by a congruent trial [cC trials]). Clearly, these results do not provide strong support for the notion of generalized inhibitory deficits in subclinical anxiety and depression. A more parsimonious explanation seems to be that increased anxiety is linked to a generally more cautious approach to the task, as expressed by increased RTs, whereas increased depression is linked to a more careless approach, as expressed by decreased RTs when the immediately preceding context has provided congruent (i.e., conflict-free or “trustworthy”) information.

Although the existing literature on inhibitory control frequently focuses on behavioral adjustments following conflicting information (e.g., Egner, 2008), adjustments following conflict-free trials are, in fact, more commonly observed in response-conflict tasks. In a recent study (Schlaghecken & Martini, 2011), we demonstrated that between-trial behavioral adjustments in a variety of response-conflict paradigms can be modeled by a mechanism of context-dependent mirror-symmetrical “tightening” and “relaxing” of the visuo-motor system’s responsiveness, rather than by a mechanism of selective conflict detection and adjustment. According to this model, if after a congruent, conflict-free trial the system relaxes too much, its responsiveness will increase to an extent that even task-irrelevant distractor information can cause response execution. If the required response is the same as the one triggered by the distractor (congruent trial), this will merely result in very fast responses. However, if the required response differs from the one triggered by the distractor (incongruent trial), it will result in a (very fast) error. Thus if relaxing the perceptuo-motor system’s responsiveness too much after a conflict-free trial is behaviorally risky, then it seems reasonable to assume that an efficient cognitive control system would prevent such exaggerated relaxation. The present results therefore indicate a lack of efficiency or “bluntedness” (Steele et al., 2007) of cognitive control functions associated with heightened levels of subclinical depression symptoms.

In this context it is also worth noting that neither anxiety nor depression levels affected low-level inhibition (as measured by the NCE). This is of particular interest in the context of recent results regarding the NCE in normal aging. It is generally accepted that depression and normal aging share certain neurophysiological characteristics, such as decreased dopamine and serotonin receptor density in regions asso-ciated with both low level (basal ganglia) and high-level (ACC and dlPFC) inhibitory control (e.g., Kaasinen et al., 2000). As older participants consistently fail to produce NCEs at the masked prime-target interval employed in the present experiment (Maylor et al., 2011; Schlaghecken & Maylor, 2005), one would have expected to see a similar trend in the more depressed participants. This was not the case, suggesting that subclinical levels of depression do not mimic an aging brain (at least not with respect to inhibitory perceptuo-motor control).

### Relationship of the present findings to previous studies of inhibitory control in anxiety and depression

Although clinical levels of anxiety and depression are generally believed to be associated with inhibitory deficits (e.g., Channon & Green, 1999; Kaiser et al., 2003), the evidence that the same is true for subclinical levels of anxiety and depression symptoms is rather sparse. Regarding subclinical anxiety, inhibitory deficits have usually been obtained in affective response conflict tasks (e.g., Schrooten & Smulders, 2007; Sehlmeyer et al., 2010) and in response conflict tasks requiring attentional control (e.g., Ansari & Derakshan, 2010, 2011; Avila & Parcet, 2002). Together with these studies, the present findings confirm the view that at least at subclinical levels, anxiety-related deficits in cognitive control reflect situation-specific (particularly threat-specific) attentional dysregulation or over-vigilance (e.g., Öhman & Mineka, 2001), rather than a non-specific deficit in perceptuo-motor inhibition.

Regarding subclinical depression, the existing evidence for an inhibitory deficit is somewhat mixed. For instance, Pizzagalli et al. (2006), measuring inhibitory control in a non-affective Eriksen flanker task, found that participants with elevated levels of depressive symptoms failed to show post-error adjustment in the form of increased accuracy following an incorrect response. However, depressive symptoms did not modulate the Flanker congruency effect, the Gratton effect, or post-error slowing (PES). Holmes and Pizzagalli (2007) observed the same pattern of results in a Simon task, but only when participants had been given (fake) negative feedback.^9^ In contrast, the present experiment found both larger Simon effects and larger post-error adjustments with higher levels of depression. There are various methodological differences between those studies and the present one, from different stimuli (colored circles and squares vs. arrows) to different inter-trial intervals (2.3-3.3 s vs. 1.46 s), to different probabilities of congruent and incongruent trials (biased towards congruent in the Holmes and Pizagalli study vs. equal probabilities in the present experiment), any of which might have affected the different patterns of results. However, taken together, the overall picture emerging is not one of sub-clinical depressive symptoms being strongly associated with perceptuo-motor inhibitory deficits.

Perhaps the most interesting aspect of the present study is the finding that to the extent that heightened anxiety versus heightened depression symptoms affect behavioral control at all, they do so in contrasting ways. Liotti and Tucker (1995; as described in Liotti & Mayberg, 2001) proposed that anxiety mostly affects ventral cortico-limbic structures including the inferior temporal and the orbitofrontal cortex, assumed to support object processing and focused attention, whereas depression mostly affects dorsal cortico-limbic structures including the dlPFC and inferior parietal cortex, assumed to be involved in spatial processing and the control of externally directed attention (see also Liotti et al., 2000). In the present study, requiring non-affective spatial processing, increased levels of subclinical anxiety and increased levels of subclinical depression were associated with specific, non-overlapping impairments (increased RTs but unaffected inhibitory effects vs. unaltered RTs but increased Simon effects). Such a dissociation appears to be in line with a two-systems model. However, further research is required to determine the exact extent to which “over-cautiousness” versus “over-relaxation” of the perceptuo-motor system are associated with increased levels of subclincial anxiety and depression, respectively.

## Footnotes

1 Our notion of inhibitory control here is most closely related - though not identical - to the concept of suppression of a prepotent response tendency (e.g., Miyake et al., 2000). However, the neural processes underlying inhibitory effects are not yet fully understood, as even phenomena generally assumed to reflect cognitive and/or motor inhibition might, in fact, be comprised of inhibitory (reducing neural excitability) and excitatory (increasing neural excitability) components (e.g., Schlaghecken & Martini, 2011). We will return to this issue briefly in the Discussion.

2 However, a substantial NCE-like effect occurs at a disproportio-nal delay in most of these participants (Maylor et al., 2011).

3 One might note that Boy et al. (2010) reported larger NCEs in participants with lower levels of gamma-amino-butyric-acid (GABA). This might be considered of relevance in the current context, as GABA levels are generally found to be lowered in anxiety and depression (for a review, see Kalueff & Nutt, 2007). However, Boy et al.’s finding was specific for the supplementary motor area (SMA) of the frontal lobes, an area that is not implicated in anxiety or depression disorders (Kaleuff & Nutt, 2007). Consequently, we do not see a reason to predict larger NCEs for participants with higher anxiety and depression scores.

4 If interference suppression breaks down completely, an incor-rect response will be given on the majority of incongruent trials.

5 In this paradigm, masked primes do not produce reliable Gratton effects (Schlaghecken, Refaat, & Maylor, 2011), and prime-compatibility of the previous trial was therefore not considered in the present study.

6 Note that according to the first account, large PES indicates strong anticipatory cognitive control, whereas according to the latter two, it indicates weak online inhibitory control (overcoming erroneous processes) and weak attentional control (withdrawing attention from a past event), respectively. To the extent that all three processes might play a role in generating PES, the value of this effect as an indicator of inhibitory strength is somewhat limited.

7 As Simon effects are already ratios, Gratton effects could have been calculated as differences between these without confounding effect magnitude with overall RTs. However, we felt that it would be preferable to present all effects in the same format. Statistical analysis of difference-Gratton effects produced a similar pattern of results to the one reported here.

8 However, inspections of Figure 2 suggests that most (possibly all) of these effects might be due to floor effects, as appreciable numbers of errors only occurred on cI (incongruent following congruent) trials when the previous response had been correct.

9 In the same study, however, elevated levels of depression were associated with a reduced Gratton effect - independent of feedback - in a Stroop task.
